# Gene-body CG methylation and divergent expression of duplicate genes in rice

**DOI:** 10.1038/s41598-017-02860-4

**Published:** 2017-06-01

**Authors:** Xutong Wang, Zhibin Zhang, Tiansi Fu, Lanjuan Hu, Chunming Xu, Lei Gong, Jonathan F. Wendel, Bao Liu

**Affiliations:** 1 Key Laboratory of Molecular Epigenetics of the Ministry of Education (MOE), Northeast Normal University, Changchun, 130024 P. R. China; 2 Ecology, Evolution and Organismal Biology, Iowa State University, Ames, IA United States; 3 Department of Agronomy, Purdue University, West Lafayette, USA

## Abstract

Gene and genome duplication fosters genetic novelty, but redundant gene copies would undergo mutational decay unless preserved via selective or neutral forces. Molecular mechanisms mediating duplicate preservation remain incompletely understood. Several recent studies showed an association between DNA methylation and expression divergence of duplicated genes and suggested a role of epigenetic mechanism in duplicate retention. Here, we compare genome-wide gene-body CG methylation (BCGM) and duplicate gene expression between a rice mutant null for OsMet1-2(a major CG methytransferase in rice) and its isogenic wild-type. We demonstrate a causal link between BCGM divergence and expression difference of duplicate copies. Interestingly, the higher- and lower-expressing copies of duplicates as separate groups show broadly different responses with respect to direction of expression alteration upon loss of BCGM. A role for BCGM in conditioning expression divergence between copies of duplicates generally holds for duplicates generated by whole genome duplication (WGD) or by small-scale duplication processes. However, differences are evident among these categories, including a higher proportion of WGD duplicates manifesting expression alteration, and differential propensities to lose BCGM by the higher- and lower-expression copies in the mutant. Together, our results support the notion that differential epigenetic marking may facilitate long-term retention of duplicate genes.

## Introduction

Polyploidy, or whole genome duplication (WGD), represents a major mechanism for enhancing organismal gene content and diversification. WGD is a recurrent feature in the evolutionary histories of both plants and animals, and has played a particularly pervasive role in the diversification of angiosperms^1^. Over periods ranging from thousands to millions of years, newly duplicated genomes become partially to mostly diploidized by multiple evolutionary genomic processes^2^. In addition to WGD events, smaller-scale and single gene-based duplications are common in all plant genomes analyzed to date^3–5^. Consequently, duplicate genes are abundant in the genome of all angiosperms, reflecting a balance between WGD and single-gene-based duplication events, and the subsequent extensive yet never-complete diploidization. An important aspect of these dynamics is that of differential or biased retention of duplicates, and how this differs for genes derived from these two primary duplication mechanisms^6–8^. Molecular and evolutionary mechanisms that underlie gene retention include considerations of dosage balance^9–11^, quaternary structure and functional constraints^12^ and gene expression levels^5, 9, 13–16^.

Down-regulation of aggregated or total expression level for any pair of gene duplicates may be achieved by lowering expression of one copy and/or concomitant reduction of expression levels of both copies. Multiple molecular mechanisms may underpin these changes, including *cis*-regulatory divergence at the nucleotide sequence level^17^, structural changes including physical loss of exons^18^, and epigenetic modifications. With respect to the latter, accumulating evidence in recent years from diverse organisms indicates that divergence in DNA methylation, i.e., differential adding and/or maintenance of a methyl group to CG (primarily) cytosines to form 5-methylcytosine, is common for duplicated copies of a given gene pair, and that this is correlated with differential expression. For example, in multiple human tissues DNA methylation divergence occurs in promoter regions following gene duplication, increases with evolutionary time, and correlates with tissue-specific expression^19^. In rice, changes in both pattern and level of gene body methylation correlate with expression divergence of duplicates, although the correlation directions (positively or negatively) are different for duplicates of different origins or duplication models^20^. In soybean, WGD genes that are more gene-body CG-methylated were found to show higher levels of expression, and were more likely to be retained as duplicates^21^. In cassava, a strong positive correlation was observed between gene body methylation and expression of duplicated genes following WGD^22^. Notably, gene pairs with more divergent gene body methylation and expression differences are enriched for specific functional classes that likely are under human selection during domestication and later genetic improvement^22^. Nevertheless, all evidence obtained to date on the relationships between divergence in DNA methylation and expression of gene duplicates is correlative by nature, rendering a causal link between the two phenomena uncertain due to lack of experimental validation.

Rice (*Oryza sativa* L.) has experienced at least two ancient WGD events^23, 24^. Although 70 million years have elapsed since the last WGD episode (ρ) in rice^1^, its genome retains many duplicated genes that are apparent legacies of this WGD^25^. Apart from those of WGD origin (often as duplicated chromosomal segments), duplicates derived from single gene-based duplication mechanisms (often as small scale duplications) can be further classified into several distinct types according to the physical distance between duplicates, i.e., tandem duplicates, proximal duplicates, and transposed duplicates^20^. These different classes of duplicates were found to have distinct body methylation patterns, as well as heterogeneous relationships with expression^20^; accordingly, gene body methylation might play an important role in differential retention of duplicate genes in plants^26, 27^.

Here, we take a mutation-based approach to study the relationship between gene body methylation and expression of duplicate genes. Specifically, we took advantage of the availability of methylome and transcriptome data in a null mutant of the major CG methytransferase, *OsMet1-2*, in the standard rice genotype Nipponbare, which we generated previously^28^. The *OsMet1-2* null mutant shows a global loss of *ca*. 75% CG methylation compared with its isogenic wild type (WT), when all sequences are considered together^28^, and *ca*. 91% CG methylation loss in gene bodies^29^. Importantly, this loss of CG methylation in the *OsMet1-2* null mutant does not affect CHG and CHH methylation^28^. This dataset thus provides a tractable experimental system to explore causal links between the two molecular phenotypes, CG methylation and expression, in relation to duplicate genes in rice. In light of the previous finding that gene body methylation is variably associated with duplicates of different origins, i.e., WGD, tandem, proximal and transposed^20^, we also investigated if these different types of duplicates respond similarly or differentially to the loss of CG methylation and the attendant impacts on duplicate expression.

## Results

### Gene duplicates of different origins in the rice genome have distinct evolutionary histories

**Figure 1:**
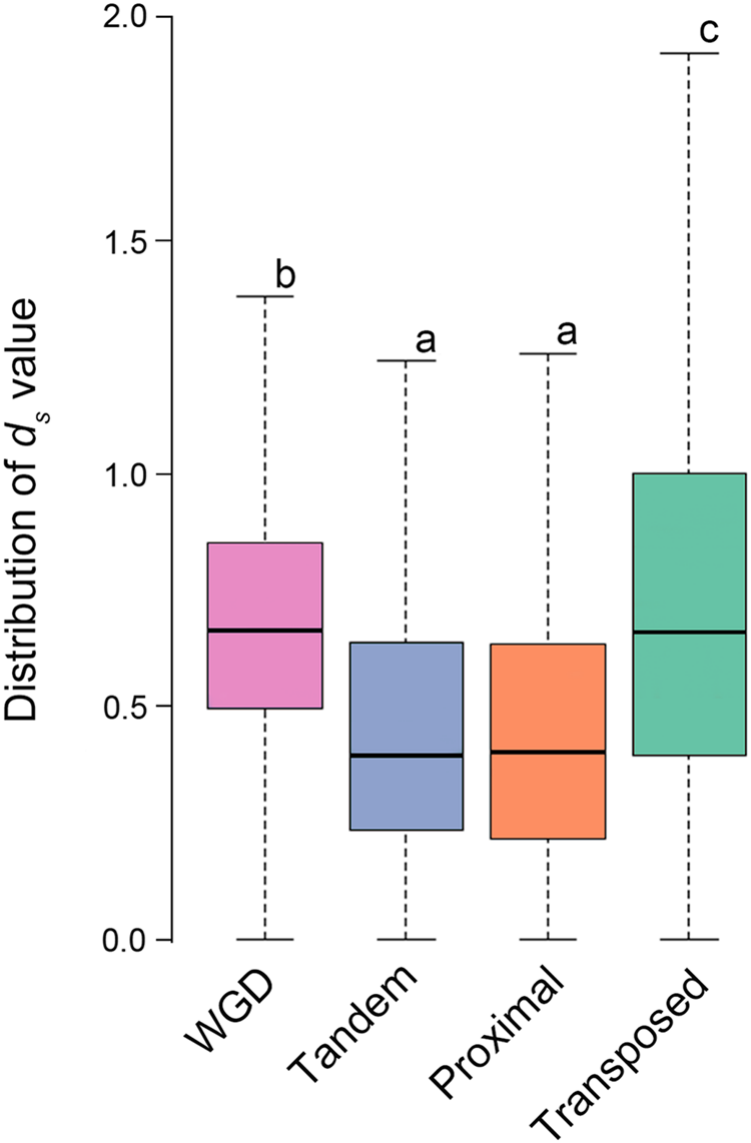
Box-plots showing distribution of the synonymous (*d*_*S*_) substitution rates for each of the four different categories of duplicate genes. The *y* axis shows the distribution of *d*_*S*_ values in the four categories of duplicates. The results show significantly different *d*_*S*_ distributions in some but not all the pairwise comparisons among the different categories of duplicates, according the Kolmogorov-Smirnov test (*p* values < 2e-16). Boxblots with different letters indicate statistically different *d*_*S*_ distributions.

In addition to duplicates derived from the last WGD *ca*.70 MYA^30^, duplicates generated by single gene-based mechanisms are also abundant in the rice genome. These were classified into distinct types according to their likely mode of duplication and physical distance between the duplicates, i.e., tandem, proximal and transposed duplicates^20^. For the purpose of exploring whether DNA methylation has played similar or different roles in regulating expression of duplicated genes having different origins, we first identified and classified the duplicates according to criteria defined previously^20^ based on the updated version of the annotated rice reference genome (MSU7, detailed in Materials and Methods). We identified 4871, 9028, 3827 and 3281 distinct genes for the WGD, transposed, proximal and tandem duplication categories, respectively, corresponding to 2961, 5697, 2171 and 1862 gene pairs, respectively (Table 1). All of these genes have high quality transcriptome and methylome data from the same tissue (seedling leaf) in the standard laboratory wild type (WT) rice cultivar (Nipponbare) and its isogenic null mutant of the *OsMet1-2* gene^28^. To investigate whether the duplicate genes of different origins have differential evolution histories, we calculated the synonymous (*d*_*S*_) substitution rates of duplicates within each of the identified genes. Significantly different *d*_*S*_ distributions were observed among some of the different types of duplicates (ANOVA, *p* values < 2e-16, Fig. 1). Specifically, *d*_*S*_ of the WGD duplicates ranged from 0.5 to 0.85, significantly different from those of the other three categories, tandem (0.23 to 0.63), proximal (0.22 to 0.64) and transposed duplicates (0.4 to 1.0) (Fig. 1). Among the later three categories, *d*_*S*_ of tandem and proximal are statistically equal, but both are different from *d*_*S*_ for transposed duplicates (Fig. 1). The relatively higher *d*_*S*_ values of the WGD duplicates are consistent with the ρ WGD event (occurred~70 MYA) in rice^24, 30^, while the other classes of gene duplicates (except transposed duplicates) are younger (Fig. 1). We found the *d*_*S*_ distributions of transposed duplicates to be broader than those of the other categories (Fig. 1), suggesting that the evolutionary footprint of transposed duplicates remains evident for a longer period of time than for proximal and tandem duplicates. In view of the distinct evolutionary histories of the different categories of gene duplicates, we wished to test whether and to what extent their methylation states would be different, whether null mutation of the major CG-methytransferase (*OsMet1-2*) would cause similar or different loss of CG methylation, and the impacts on total and copy-specific expression of the duplicates in each category.

**Table 1:** Studied duplicate genes in rice originating from different duplication mechanisms.

Duplication category	Number of duplicate pairs	Number of distinct genes
WGD	2961	4871
Transposed	5697	9028
Proximal	2171	3827
Tandem	1862	3281

### Duplicate genes of different origins all show substantial loss of gene body CG methylation in the *OsMet1-2* null mutant

**Figure 2:**
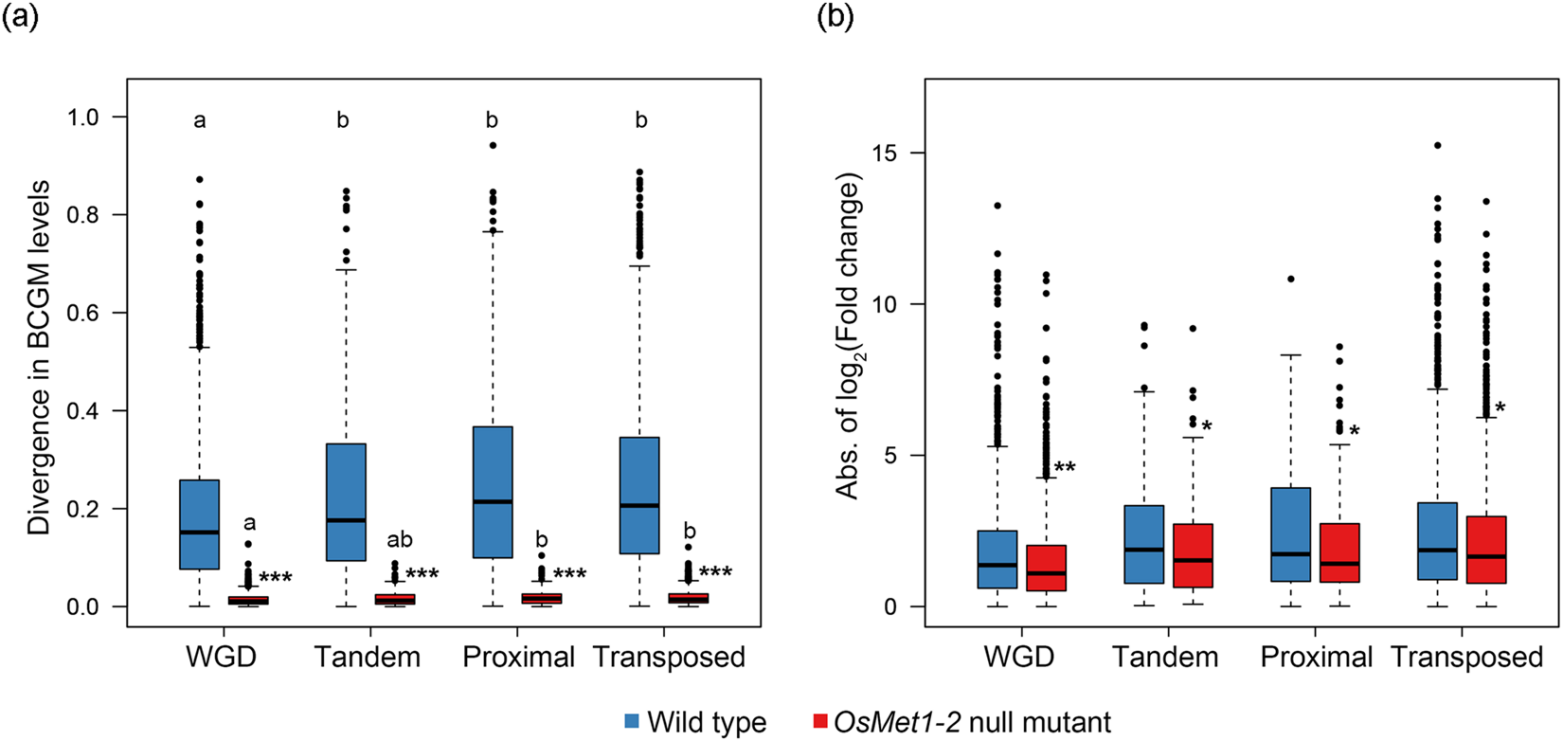
Divergence in body CG methylation (BCGM) level (**a**) and expression (**b**) between duplicated copies of each of the four different categories of duplicates with CG-only methylation in WT rice and the *OsMet1-2* null mutant. The *y* axis in (**a**) denotes divergence in BCGM levels between duplicated copies of each duplication category. Pairwise comparisons showed significant differences in between-copy BCGM divergence among some (indicated by different small letters) but not all (indicated by the same small letters) of the four duplication categories in WT (ANOVA and Tukey’s honestly significant different (HSD) test, *p* < 2.42e-14). Significant reduction of between-copy divergence in BCGM was detected in mutant versus WT in all duplication categories (Kolmogorov-Smirnov test, *p* values < 0.001). The *y* axis in (**b**) shows absolute value of fold changes of between-copy expression levels of each duplication category in WT and mutant. Significant reduction of between-copy expression difference in mutant versus WT was detected for all categories of duplicated genes (Kolmogorov-Smirnov test, *p* values < 0.05).

It is known that methylation of individual cytosine bases, i.e., methylated cytosines, is metastable across genotypes, individuals, tissues/developmental stages, and even different environmental conditions^31–34^. Thus, to obtain more robust results that are likely evolutionarily relevant, we adopted the strategy of a previous study in tabulating methylome data as regional CG methylation levels^20^ rather than assessing methylation differences of individual cytosine bases. Also, because CG methylation of coding regions (gene body CG methylation) is more evolutionarily conserved than methylation of other genomic regions in plants^26, 27^, we primarily focused on CG methylation level of gene bodies, i.e., body CG methylation (BCGM) levels. We tabulated BCGM levels for all identified gene duplicates of different origins based on the whole-genome bisulfite sequencing-generated methylome data for both WT and the *OsMet1-2* mutant^28^, and defined 2,805 gene duplicates with CG-only methylation and 4,460 gene duplicates with all-context (CG, CHG and CHH) methylation (defined in Methods). We found that nearly all the duplicates with CG-only methylation showed significant loss of BCGM at least in one copy of a given gene pair in the mutant (Table 2). Similarly, 73–99% of duplicates with all-context methylation showed the same trend (Supplementary Table S1). These results are consistent with our prior results of massive loss of CG methylation at gene body regions in the mutant^28, 29^. Further, we investigated BCGM level divergence between the duplicates in WT and mutant, respectively, for both duplicates with CG-only methylation and duplicates with all-context methylation. For duplicates with CG-only methylation, BCGM level divergence between duplicate copies of the four categories in both WT and the mutant showed the same trend of transposed ≈ proximal ≈ tandem > WGD (ANOVA and Tukey’s honestly significant different (HSD) test, *p* < 2.42e-14; Fig. 2a). For duplicates with all-context methylation in WT, this trend was less clear, but again the trend was similar between WT and the mutant, and all four categories of duplicates varied in extents of BCGM level divergence between duplicated copies (K-S test, *p* values < 2e-16; Supplementary Fig. S1a). In another word, BCGM level divergence was dramatically reduced in all four categories of duplicates irrespective of CG-only methylation or all-context methylation in the mutant relative to WT (K-S test, *p* values < 2e-16; Fig. 2a; Supplementary Fig. S1a), but the decrements were significantly smaller in the later (Supplementary Fig. S2) than in the former (K-S test, *p* values < 2e-16). This observation may implicate a fortifying role by non-CG methylation (CHG methylation in particular) in maintaining CG methylation in the mutant, as CHG methylation showed little reduction in the mutant versus WT, and the basal level of CHH methylation is intrinsically low in WT rendering its loss in the mutant^28, 29^ (Supplementary Fig. S3) likely inconsequential.

**Table 2:** Statistics of duplicates with CG-only methylation that showed loss of BCGM (body CG methylation) in the *OsMet1-2* mutant, and the proportion that showed changed expression levels in the mutant.

Duplication category	No. of duplicates with CG-only methylation identified	No. and % of expressed duplicates showing decreased BCGM in mutant versus WT	No. and % of BCGM-reduced duplicates that showed changed expression level in mutant versus WT
Tandem	248	242 (97.6%)	84 (34.7%)
Proximal	245	239 (97.6%)	65 (27.2%)
Transposed	1299	1296 (99.8%)	474 (36.6%)
WGD	1013	1013 (100%)	473 (46.7%)

### Loss of gene body CG methylation in the *OsMet1-2* null mutant reduces original expression differences between duplicate copies

To investigate whether the loss of BCGM in the *OsMet1-2* mutant would affect the relative expression levels of duplicates intrinsic of WT rice, we selected 2790 duplicate gene pairs with CG-only methylation and 3839 duplicate gene pairs with all-context methylation, which were expressed (FPKM > 0.1) in at least one genotype (WT or mutant) of the studied tissue (young seedlings), and which also showed significant loss of BCGM in at least one copy of a given duplicate pair (Table 2). First, we tabulated the number of expressed gene pairs in each genotype. For duplicates with CG-only methylation, we found that, in general, more duplicate genes were expressed in the mutant than in WT (Fisher’s exact test, *p* value = 7.806e-06), a result largely attributable to gene pairs for which both copies become expressed in the mutant; this, however, was counterbalanced to an extent by the reduced numbers of gene pairs that have only one copy expressed in the mutant (Supplementary Table S2). The same trend was observed for duplicates with all-context methylation (Supplementary Table S3). These observations suggest an overall role of BCGM in repressing expression of the duplicate genes, especially in silencing one copy in WT rice, i.e., there is transcriptional activation of the silent copy upon loss of BCGM in the mutant. Second, we calculated the number of duplicate genes that showed significant differential expression between the two copies of a given gene pair, i.e., differentially expressed (DE) duplicates, in each genotype. We identified 2464 (88.3% of 2790) and 2460 (88.2% of 2790) DE duplicates with CG-only methylation in WT and mutant, respectively, and 3072 (80.0% of 3164) and 3839 (81.9% of 3164) for duplicates with all-context methylation, respectively (Supplementary Fig. S4). Although the two numbers of DE duplicates are statistically equal between WT and mutant (**binomial** exact test, *p* value = 0.88), the correlation of expression between gene duplicate copies was significantly stronger in the mutant (Pearson’s correlation, R = 0.51, *p* value < 2.2e-16, 95% confidence interval ranged from 0.48 to 0.53) than in WT (Pearson’s correlation, R = 0.43, *p* value = <2.2e-16, 95% confidence interval ranged from 0.40 to 0.46) (Supplementary Fig. S4a and S4b). In contrast, the correlation of expression between copies for duplicates with all-context methylation did not show discernible differences between WT and mutant (95% confidence interval ranged from 0.17 to 0.24 in WT and ranged from 0.21 to 0.27 in mutant, respectively; Supplementary Fig. S4c and S4d). We also compared the proportion of expression-altered duplicates in each of the four duplicate categories in the two genotypes. Results indicated that for duplicates with CG-only methylation, WGD duplicates showed the highest percentage (*ca*. 47%) of altered expression levels as a result of loss of BCGM in the mutant, followed by the tandem and transposed duplicates (~35% and ~37%, respectively), while the proximal duplicates showed the least percentage (~27%) of expression-altered duplicates (Fisher’s exact test, *p* < 0.01); for duplicates with all-context methylation, WGD duplicates also showed the highest percentage (47.5%) of altered expression levels as a result of loss of BCGM in the mutant, but all the rest three categories of duplicates showed more or less the same proportions (*ca*. 42%) of expression-altered duplicates (Table 2; Supplementary Table S1).

Next, we compared the extent of between-copy expression difference among the four different categories of duplicate gene pairs in WT and mutant, respectively. We found that for duplicates with CG-only methylation, both genotypes showed the same trend with regard to the extent of between-copy expression difference among the four duplicate categories, that is, WGD was the smallest while the other three categories did not differ from each other (ANOVA and Tukey’s honestly significant different (HSD) test, *p* < 2e-16 for both genotypes; Fig. 2b). Compared with WT, duplicates of all four categories showed markedly reduced between-copy expression difference in the mutant (K-S test, *p* value < 0.05; Fig. 2b). For duplicates with all-context methylation, only two of the four duplicate categories, i.e., proximal and transposed, showed statistically significant reduction of between-copy expression difference in mutant versus WT (Supplementary Fig. S1b), which contrasted with situation for the duplicates with CG-only methylation (Fig. 2b), mentioned above.

We then directly computed the correlations (Pearson’s correlation) between BCGM divergence and expression divergence in WT for each of the four categories of duplicates with CG-only methylation and duplicates with all-context methylation, respectively. For duplicates with CG-only methylation, we found that significant positive correlations existed between the two molecular phenotypes for all four categories of duplicates (Supplementary Fig. S5a). By contrast, for duplicates with all-context methylation, the correlations were either reduced (categories of WGD and transposed) or abolished (categories of tandem and proximal) (Supplementary Fig. S5b). These observations suggest that non-CG methylation of duplicates blurred the relationship between BCGM and expression either directly or indirectly, possibly via their fortifying roles in maintaining CG methylation differences between duplicates when the major CG methytransferase was nonfunctional, as aforementioned.

### Gene body CG methylation is causally linked to expression of duplicate gene copies

**Figure 3:**
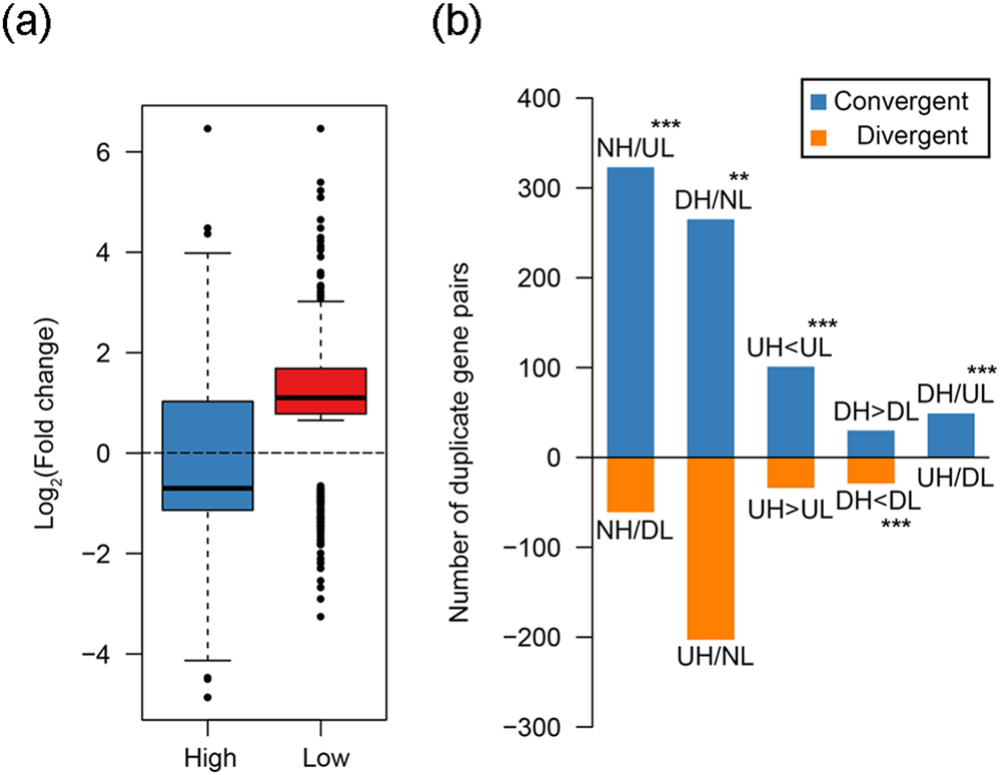
Changes in expression by the higher and lower expression copy-groups, respectively, of all identified rice duplicate genes with CG-only methylation (**a**), and convergent versus divergent expression changes between copies of the duplicate genes with CG-only methylation (**b**), due to loss of BCGM in the mutant.(**a**) The *y* axis shows fold changes of expression level between WT and mutant by the higher and lower expression copy-groups, respectively, of all identified rice duplicated genes. The dashed black line denotes fold change = 1, which divides the boxplots into two parts. Distribution of gene numbers between the upper and lower parts was tested by binomial exact test, which indicated that the two parts contained significantly different numbers of genes for both the higher expression copy group (*p* value = 0.01199) and the lower expression copy-group (*p* value < 2.2e-16). (**b**) Based on changing direction (up- versus down-regulation) and magnitude (higher versus lower) in expression by the two copies of each analyzed duplicate, the duplicated genes that showed expression differences between WT and mutant can be categorized into10 distinct groups, which can be combined into two classes according to consequences of the changes with respect to reducing or augmenting expression differences between duplicate copies, i.e., convergent and divergent (see main text for details). The *y* axis shows the number of duplicate genes in each of the 10 groups. A binomial exact test was performed to test for statistical differences in convergence versus divergence in each comparison. Aasterisks denote statistical significance: *, ** and *** are *P* values < 0.05, 0.01and 0.001, respectively.

The foregoing results documented that loss of BCGM in the *OsMet1-2* mutant reduced expression difference between duplicated gene copies. It remains unclear whether the two duplicated copies were similarly or differentially affected by loss of DNA methylation. To investigate this issue, we first divided the duplicate gene copies of all four categories into two groups (respectively for duplicates with CG-only methylation and duplicates with all-context methylation), i.e., higher and lower expression copy-groups in WT, and then interrogated the trend of expression changes of each copy-group upon loss of BCGM in the mutant. Results showed that, for both duplicates with CG-only methylation and duplicates with all-context methylation, significantly more genes in the higher expression copy-group displayed down-regulation in the mutant (binomial exact test, *p* value = 0.01199 for duplicates with CG-only methylation and 5.987e-12 for duplicates with all-context methylation), whereas more genes in the lower expression copy-group showed up-regulation (binomial exact test, *p* value < 2.2e-16 for duplicates with CG-only methylation and 6.738e-13 duplicates with all-context methylation) (Fig. 3a; Supplementary Fig. S6a). Though mechanistically mysterious, this observation is intriguing in that it suggests the relationship between BCGM level and expression of the duplicated genes can be bidirectional at the level of duplicated copies. That is, BCGM of the higher expression copies is more likely enhancing expression, while BCGM of the lower expression copies tends to repress expression.

In principle, loss of methylation in the mutant can lead to either the same or different directionality (up- versus down-regulation) and magnitude (higher- versus lower) of expression changes. Using this framework, we categorized all the analyzed duplicate gene pairs into 10 groups respectively for those with CG-only methylation and those with all-context methylation, and listed the number and percentage of each group in Tables S4 and S5. These were: (*i*) no change in the higher-expression copy and up-regulation in the lower-expression copy (NH/UL); (*ii*) no change in the higher-expression copy and down-regulation in the lower-expression copy (NH/DL); (*iii*) down-regulation in the higher-expression copy and no change in the lower-expression copy (DH/NL); (*iv*) up-regulation in the higher-expression copy and no change in the lower-expression copy (UH/NL); (*v*) up-regulation in both the higher- and lower-expression copies but the changed magnitude of the former was smaller than the later (UH < UL); (*vi*) up-regulation in both the higher- and lower-expression copies but the changed magnitude of the former was larger than the later (UH > UL); (*vii*) down-regulation in both the higher- and lower-expression copies but the changed magnitude of the former was larger than the later (DH > DL); (*viii*) down-regulation in both the higher- and lower-expression copies but the changed magnitude of the former was smaller than the later (DH < DL); (*ix*) down-regulation in the higher-expression copy and up-regulation in the lower-expression copy (DH/UL); (*x*) up-regulation in the higher-expression copy and down-regulation in the lower-expression copy (UH/DL). Collectively, compared with WT, these changes may result in either convergent expression between the duplicate gene copies, i.e., reduction of inter-copy expression difference, or divergent expression, i.e., augmentation of inter-copy expression difference, in the mutant. Specifically, groups (*i*), (*iii*), (*v*), (*vii*) and (*ix*) would reduce between-copy expression differences while groups (*ii*), (*iv*), (*vi*), (*viii*) and (*x*) would augment between-copy expression differences. We observed the following in both duplicates with CG-only methylation and duplicates with all-context methylation: first, more duplicate genes showed expression change in one copy only (i.e., groups *i–iv*) than those showing changes in both copies (i.e., groups *v*–*x*) in the mutant (binomial exact test, *p* values < 2.2e-16, Fig. 3b; Supplementary Fig. S6b); second, except for the both-copy down-regulation groups, i.e., (*vii*, DH > DL) and (*viii*, DH < DL), there were significantly more duplicate genes producing convergent expression than those producing divergent expression (binomial exact test, *p* values < 0.001, Fig. 3b; Supplementary Fig. S6b) in the mutant. This result suggests that BCGM predominantly mediates divergent expression of duplicate copies in WT rice for all categories of duplicates (Tables S4 and S5).

To validate the RNA-seq data independently, we conducted qRT-PCR analysis for 10 randomly selected duplicate gene pairs (Supplementary Table S6). Results indicated that all 10 duplicates showed between-copy expression differences (Supplementary Fig. S7) that are largely consistent with, and hence validating, our RNA-seq-based analysis. Moreover, qRT-PCR results of these 10 gene pairs indicated the higher expression copies for six gene pairs showed down-regulation in the mutant, while the lower expression copies in all 10 genes showed up-regulation in the mutant, in accordance with the general opposite relationships between BCGM and expression by the higher expression-group and lower expression-group when all the analyzed duplicates are considered (Fig. 3a; Supplementary Fig. S6a).

We further scrutinized whether the lower- or higher-expression copy for a given gene pair of each of the four duplication categories would show equal or different propensities to lose BCGM in the mutant. For duplicates with CG-only methylation, we found that the higher-expression copies showed greater loss of BCGM than the lower-expression copies in all four duplication categories in the mutant (K-S test, *p* value < 0.01; Supplementary Fig. S8a). For duplicates with all-context methylation, however, no consistent difference between the higher- versus lower-expression copies was found, instead, which varies among the duplication categories (Supplementary Fig. S8b).

**Figure 4:**
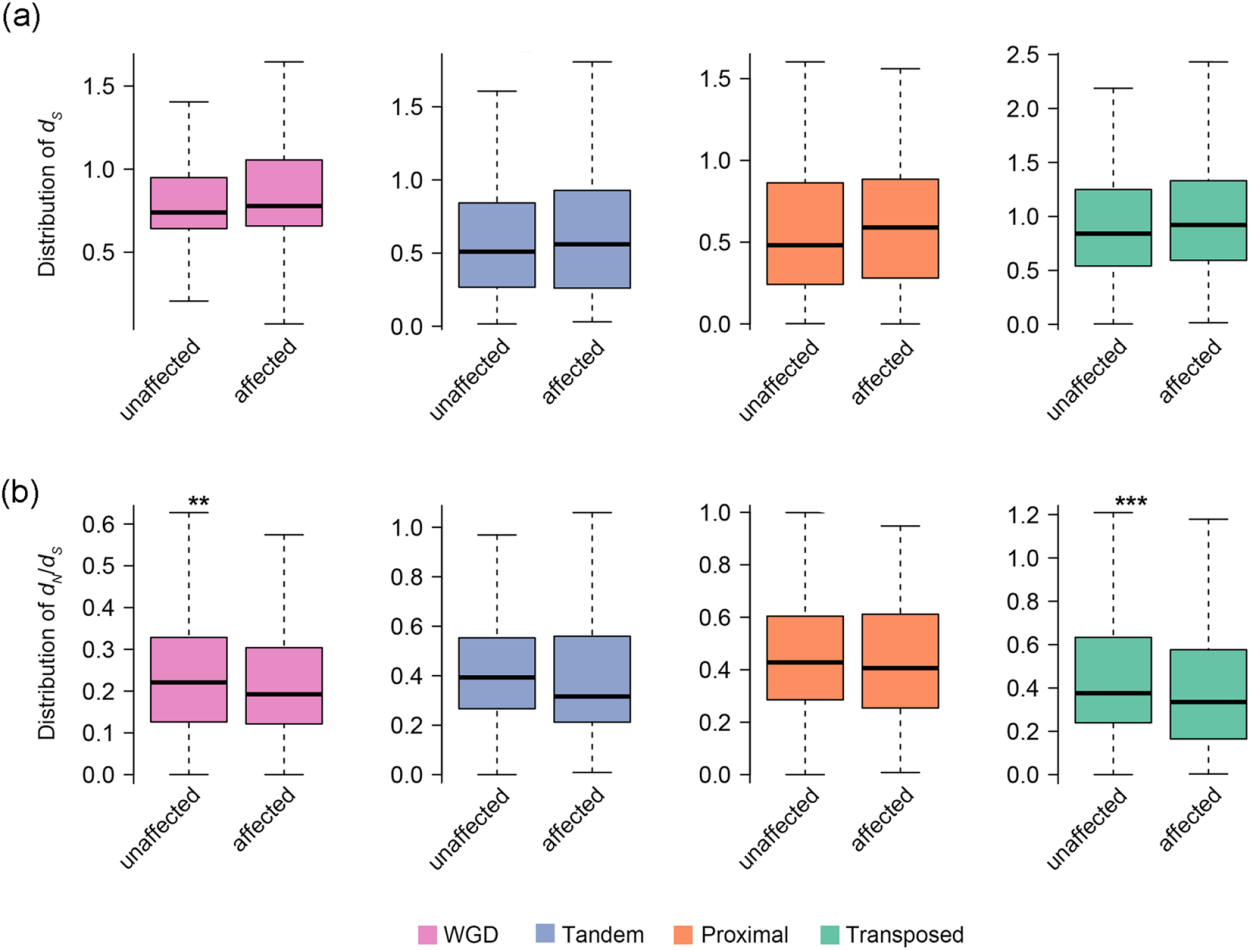
Comparison of distributions of *d*_*S*_ (**a**) and *d*_*N*_ */d*_*S*_ (**b**) between expression-unaffected and -affected duplicates of each of the four categories with CG-only methylation due to loss of BCGM. The Kolmogorov-Smirnov test was conducted for statistical significance. There were no significant changes in *d*_*S*_ distribution for any duplication category (K-S test, *p* values > 0.05), but the ranges of *d*_*N*_ */d*_*S*_ distribution were significantly larger in expression-unaffected duplicates than expression-affected duplicates (K-S test, *p* values < 0.01) in the WGD and transposed duplicates.

Finally, we explored whether the differential distribution of *d*_*S*_ among the duplicate categories is related to the effects of BCGM on expression. For both duplicates with CG-only methylation and duplicates with all-context methylation, we did not find significant changes in *d*_*S*_ distribution between the expression-affected and expression-unaffected genes in a given duplication category (K-S test, *p* values > 0.05; Fig. 4a; Supplementary Fig. S9a), suggesting expression of younger and older gene duplicates were similarly influenced by BCGM. We also tested whether the expression-affected and -unaffected duplicates due to loss of BCGM might have been subjected to different intensities of selective constraints. We analyzed the distribution of *d*_*N*_/*d*_*S*_ ratio in all types of duplicates. We found that the ranges of *d*_*N*_/*d*_*S*_ distribution were larger in expression-unaffected than affected duplicates in the WGD and transposed categories of duplicates with CG-only methylation, and in the proximal and transposed categories of duplicates with all-context methylation, respectively. (K-S test, *p* values < 0.01; Fig. 4b; Supplementary Fig. S9b). These results suggest that the expression-affected duplicates due to loss of BCGM are more likely under stronger selective pressure than the expression-unaffected duplicates. This is consistent with the idea that epigenetic markers like DNA methylation *per se* may constitute substrates for Darwinian selection^35^, and which is likely more so for epigenetic marks that are functionally relevant, i.e., that affect gene expression, and by extension, phenotypes.

## Discussion

Cyclical whole genome duplication (WGD) has been established as a prominent feature in angiosperm evolution^1, 36–42^. This, together with the recurrent occurrence of the various types of single gene-based duplications^3–5^, makes genomes of all present-day “diploidized” higher plants mosaics of single-copy (singleton) and duplicated genes and genomic regions. Therefore, investigating the evolutionary roles of gene and genome duplication in general and fate of duplicate genes in particular^7^ is essential to further our understanding of genome and organismal evolution of plants and crops^5, 43, 44^.

For more than 75 years^45, 46^, evolution by gene duplication has been increasingly accepted as a driving force for the origin of functional innovation and organismal complexity^47, 48^. Theory predicts that loss-of-function mutations (degenerative mutations) should be much more frequent than gain-of-function mutations (beneficial mutations). Thus, pseudogenization leading to loss of one of the copies should be the most frequent outcome following gene duplication irrespective of the models for their genesis^49^. Counterbalancing this mutational decay are several complementary forces, including various forms of “subfunctionalization”^50^, whereby the ancestral function is partitioned (i.e., subfunctionalized) into the duplicated copies such that both become essential and hence are selectively retained, and neofunctionalization, whereby one duplicate copy evolves a new and essential function^50–52^. The molecular mechanisms that underlie the evolvability and retention of duplicate genes are thus of fundamental interest.

It was proposed more than a decade ago that epigenetic mechanisms, especially DNA methylation, may play an important role in the evolution of duplicate genes^53^. These authors proposed that if the duplicated copies undergo differential epigenetic silencing in a tissue- and/or developmental stage-complementary manner, then duplicates should be under selective constraint even without subfunctionalization, in the sense of functional degeneration or elaboration by each copy, and hence would be protected from “pseudogenization”^53^. Similarly, Adams *et al*.^54^, demonstrated reciprocally and epigenetically silenced duplicate genes in polyploid cotton, and suggested that epigenetic protection from mutational loss could be important in evolution. Several recent empirical studies have lent further support to this idea by showing a correlation between DNA methylation and evolution of duplicate genes. For example, it was found in multiple human tissues that DNA methylation divergence at promoters (though not at gene-bodies) and evolutionary age (neutral sequence divergence) are coupled, and which correlates with expression divergence of gene duplicates^19^. A study in rice demonstrated that gene-body methylation and divergence is associated with evolutionary age of duplicates; moreover, duplicates generated by different models (e.g., WGD versus single gene-based duplications) displayed different relationships with respect to DNA methylation and evolutionary divergence^20^. In addition, cross-species comparisons of common WGD-derived paralogs in plants (e.g., between monocot rice and dicot *Arabidopsis*) indicated that body-methylation divergence positively correlates with both expression difference and genetic divergence of the paralogs^55^. The more prominent role of gene body than promoter methylation in plants (but not in animals) is not surprising as the former represents the major form of DNA methylation, is associated with gene expression, and is highly conserved over evolutionary time^26, 27, 56, 57^.

Hitherto, all evidence implicating a role of DNA methylation on expression divergence of duplicate genes is based exclusively on correlative analyses between the two molecular phenotypes^19, 20, 22, 55^. Here, we investigated the massive loss of CG body methylation (BCGM) for the different categories of duplicate genes in rice^20^ resulting from a null mutation of the major CG methyl-transferase, *OsMet1-2* ^28^, and the attendant effects on expression changes of the gene duplicates. We found that although gene duplicates originating by different modes have distinct evolutionary histories and display different extents of BCGM divergence, they all showed massive loss of methylation, which resulted in significant reduction of BCGM divergence between duplicate copies in the mutant relative to its isogenic wild type. Concomitantly, expression difference between the gene duplicates was also reduced in the mutant. Thus, by comparative analysis of methylomes and transcriptomes of WT and the mutant, our results establish a genome-wide causal link between BCGM divergence and expression difference between copies of duplicate genes having different origins in rice. Notably, duplicates originating from WGD showed the largest proportion of expression change following BCGM loss in the mutant, suggesting that retained duplicates from WGD events are dependent more heavily on BCGM than are duplicates derived by other mechanisms. This observation indicates that at least for rice, while BCGM plays an evolutionarily persistent causal role in conditioning divergent expression of all types of duplicate genes, it is different among duplicates of different origins.

Further dissection of the roles of BCGM in relation to the higher expression-copy group and the lower expression-copy group of all categories of duplicate genes has unraveled an intriguing phenomenon that has not been reported previously. That is, while the higher expression-copy group showed a significantly reduced expression in the mutant, the opposite is true for the lower expression copy-group, i.e., BCGM generally plays enhancing and repressive roles for expression level of the higher and lower expression-copy groups of duplicated genes, respectively. This is consistent with the observation that massive loss of BCGM in the *OsMet1-2* null mutant reduced expression divergence between the duplicate copies. Of note, our results have experimentally verified and extended the earlier finding that BCGM has a heterogeneous relationship with duplicate expression in rice^20^.

Divergence in expression between gene duplicates is probably a precondition for their eventual functional diversification, and is likely related to divergence time. This possibility is consistent with the hypothesis that duplicate genes and their functional redundancy can be retained by down-regulating expression of duplicated copies such that constant total expression is ensured^14^. According to this hypothesis, constant total expression for a given duplicate gene can be achieved via lower expression of one copy and compensatory higher expression by the other copy. As such, the sufficiently lower-expressing copy may no longer be under selective constraint, i.e., they are free to evolve neutrally or adaptively leading to eventual gain of a new function^16^. Indeed, a recent study in the cotton genus (*Gossypium*) has documented that expression divergence between gene duplicates is surprisingly rapid and extensive: near-complete expression-level divergence was accomplished for all studied duplicated paralogs of two cotton sister species since their common WGD^58^. Although not yet tested, the fact that a large proportion of these duplicates showed tissue and/or developmental complementary expression patterns^58^ strongly implicates an epigenetic underpinning (at least in part) for the rapid and dramatic expression divergence of the duplicates. Our observation in this study that in the methylation mutant more genes showed expression changes in one copy of the duplicates than those showing changes in both copies is consistent with the possibility that BCGM methylation divergence of duplicate copies underpins their expression divergence. Although regulatory sequence divergence between duplicates undoubtedly plays a major role during evolution for their expression divergence, epigenetic variation such as DNA methylation changes may occur at a faster rate. For example, studies in natural populations of *Arabidopsis* showed that spontaneous single methylation polymorphisms (counterpart of single nucleotide polymorphisms or SNPs) occur at least four orders of magnitude more frequently than genetic mutations, which can be either coupled with or independent of genetic changes^59, 60^. Notably, these studies have used single-seed-derived populations under the same environment. Given that DNA methylation is prone to perturbation by both biotic and abiotic stresses, and changed methylation patterns in plants are readily inherited transgenerationally^61–64^, it is conceivable that under real natural settings, the rate of methylation changes in plant populations can be much greater. It is therefore tempting to believe that duplicated gene copies would rapidly accumulate DNA methylation divergence, which contributes to their differential expression and preservation before mutation-based functional divergence (subfunctionalization and neofunctionalization) takes place.

To conclude, we demonstrate a causal link between gene body CG methylation (BCGM) divergence and expression difference of duplicated gene copies in rice. We show that the higher- and lower-expressing copies of duplicates, as separate groups, manifest broadly different responses with respect to direction of expression change subsequent to loss of BCGM resulted from null mutation of the major CG methytransferase-coding gene. A role for BCGM in conditioning expression divergence between duplicates generally holds for duplicate genes generated by whole genome duplication (WGD) or by small-scale duplication processes. However, differences are evident among these categories, including a higher proportion of WGD duplicates manifesting expression changes upon loss of BCGM, and differential propensities to lose BCGM by the higher- and lower-expression copies in the methylation-loss mutant. Together, our results emphasize the complex relationships between gene body methylation and expression evolution of duplicate genes in rice, which may facilitate long-term retention and hence functional innovation of duplicate genes.

## Methods

### Plant materials

Heterozygous seeds (FT928341) of a *Tos17* insertion mutant for the rice (cv. Nipponbare) *OsMet1-2* gene were obtained from the National Institute of Agrobiological Sciences (Tsukuba, Japan) and then selfed for five additional generations in our lab. Plants harboring homozygous mutations for this gene were obtained by immediate segregating the heterozygous plants via selfing. Shoots of 11-d-old seedlings of the mutant and its isogenic wild type (WT) were generated for DNA/RNA isolation^28^. Genome-wide bisulfite-sequencing (MethylC-seq) and RNA-sequencing were conducted as previously described^28^.

### RNA-seq data processing

Raw RNA-seq data for the *OsMet1-2* mutant and WT were produced previously (Hu *et al*.^28^) and retrieved from published data (SRP043448 at the Sequence Read Archive (SRA) database). Low quality reads (Phred < 30) were removed from the raw data using the FASTX-Toolkit^65^. All reference sequences (FASTA) and annotation files (GFF3) were from the latest MSU7.0 rice genome (ftp://ftp.plantbiology.msu.edu/pub/data/Eukaryotic_Projects/o_sativa/annotation_dbs/pseudomolecules/version_7.0/all.dir/). Cleaned data of each genotype were mapped to the reference rice genome using Tophat2^66^, with one mismatch allowed. Differential expression analysis was performed using Cuffdiff^66^, and differentially expressed genes (DEGs) were defined using a *q* value < 0.05. We defined those duplicate gene pairs as expression-affected duplicates if one copy of a given duplicate pair was significant changed in expression between WT and *OsMet1-2* mutant. We also defined differentially expressed duplicates in each genotype using the exact condition test (*q* value < 0.05) reported previously in soybean^67^.

### Methyl C-seq data processing

Whole-genome bisulfite sequencing (Methyl C-seq) data for both the *OsMet1-2* mutant and WT were retrieved from previously published datasets (SRP043447 at the SRA database)^28^. After removing low quality reads, the cleaned data were mapped using Bismark^68^. We retained for further analysis only the uniquely mapped reads and cytosine sites with ≥ 4 reads. Gene body methylation level was calculated as described^28^. Differences in methylation were statistically tested using Fisher’s exact test between the two genotypes. We defined duplicates as having differential gene body methylation duplicates if the methylation level was significantly different (*q* value < 0.05) in at least one copy between the two genotypes.

### Defining duplicates with CG only-methylation and duplicates with all-context methylation

First, we defined body-CG-methylated genes by using a previously reported criterion in *Arabidopsis thaliana* with some modifications^26^. Taking CG methylation for instance, *p*_cg_ is defined as the proportion of methylated cytosine residues at CG context across the body region for all non TE-related genes, and the binomial distribution test was used to analyze whether the body CG methylation level (BCGM) level is different from *p*_cg_. Genes whose BCGM level is not significantly lower than *p*_cg_ are defined as body-CG-methylated gene (BCGM genes). Similarly, body-CHG-methylated gene (BCHGM gene) and Body-CHH-methylated gene (BCHHM gene) were defined as above, respectively. Based on this framework, then, duplicated gene pairs containing at least one BCGM copy was called Body CG-methylated (BCGM) duplicated genes. Finally, duplicates with CG-only methylation were defined if both copies of a body CG-methylated (BCGM) duplicated gene pair was neither body-CHG-methylated (BCHGM) nor body-CHH-methylated (BCHHM). The rest of the duplicated genes were defined as duplicates with all-context methylation.

### Identification of duplicates of different origins

This was done based on criteria defined previously in rice^20^. In brief, non-TE-related genes were extracted from the rice reference genome (MSU7). Then, the all-vs-all Blastp^69^ was used to identify candidate duplicates and a gene pair that was top 5 matched and with an E-value < 10^−10^ was considered as a candidate duplicates. Then, MCscanX^70^ was performed to categorize different types of duplicates, included WGD, tandem, proximal and transposed duplicates, with default parameters. Finally, we only selected those duplicates that have methylation information in both the *OsMet1-2* mutant and WT for further analysis.

### Calculation of *d*_*S*_ and *d*_*N*_

Synonymous (*d*_*S*_) and non-synonymous (*d*_*N*_) mutations were calculated as follows: all coding region sequences and protein sequences of duplicates were pairwise aligned using the default options in MUSCLE^71^, and the alignment results were used to calculate *d*_*S*_ and *d*_*N*_ values using the ‘seqinr’ package in R^72^. As per the previous study in rice^20^, when *d*_*S*_ > 3, duplicates were excluded.

### Real-time qRT-PCR analysis

Total RNAs were independently isolated from the two genotypes under the same conditions as those for RNA-seq^28^. A set of 10 duplicated genes pairs were randomly chosen and copy-specific qRT-PCR primers were successfully designed for 10 genes (Supplementary Table S6). For each of these 10 duplicate genes, the relative expression level of the higher and lower expression copies in WT and mutant were calculated. The Student’s t-test was used to test for statistical difference in relative expression level between WT and mutant for each copy of a given duplicated gene pair.

### Statistics

All Statistical tests in this paper were performed using basic packages in R language (Version 3.3.1)^73^.

## Electronic supplementary material

Supplementary Information
