# Supplementary material for: Gene-body CG methylation and divergent expression of duplicate genes in rice
Source: Sci Rep. 2017 Jun 1;7:2675. doi: 10.1038/s41598-017-02860-4 (PMC5453933; doi:10.1038/s41598-017-02860-4)
Supplement: Supplementary file 1 — Supplementary Information [file 41598_2017_2860_MOESM1_ESM.doc]

**Gene-body CG methylation and divergent expression of duplicate genes in rice**

**Xutong Wang^1†ǂ^, Zhibin Zhang^1†^, Tiansi Fu^1^, Lanjuan Hu^1^, Chunming Xu^1^, Lei Gong^1,2^, Jonathan F. Wendel^2*^, and Bao Liu^1^*^*^***

**SUPPLEMENTAL INFORMATION**

**Table S1.** Statistics of duplicates with all-context methylation which showed loss of BCGM (body CG methylation) in the *OsMet1-2* null mutant, and the proportion that showed changed expression levels in the mutant.

| Duplication category | No. of duplicates with all-context methylation | No. & % of expressed duplicates showing reduced BCGM in mutant versus WT | No. & % of BCGM-reduced duplicates that showed changed expression level in mutant versus WT |
| --- | --- | --- | --- |
| Tandem | 595 | 437 (73.4%) | 184 (42.1%) |
| Proximal | 971 | 730 (75.2%) | 292 (40.0%) |
| Transposed | 2391 | 2175 (91%) | 906 (41.7%) |
| WGD | 503 | 497 (98.8%) | 236 (47.5%) |

**Table S2.** Statistics of duplicates with CG-only methylation that were expressed in either or both WT and the *OsMet1-2* null mutant.

| Genotype | Not-expressed | Copy #1 expressed | Copy #2 expressed | Both-copy expressed |
| --- | --- | --- | --- | --- |
| WT | 34 | 279 | 133 | 2344 |
| Mutant | 5 | 215 | 99 | 2471 |

Note: 1. FPKM > 0.1 is the cutoff value for expressed genes.

2. Fisher’s exact test was applied to test for differences in the overall numbers of expressed duplicated genes in WT versus mutant, which showed that significantly more duplicated genes were expressed in the mutant than in WT (*p* value = 7.806e-06).

**Table S3.** Statistics of duplicates with all-context methylation that were expressed in either or both WT and the *OsMet1-2* null mutant.

| Genotype | Not-expressed | Copy #1 expressed | Copy #2 expressed | Both-copy expressed |
| --- | --- | --- | --- | --- |
| WT | 328 | 1232 | 487 | 1792 |
| Mutant | 91 | 1170 | 504 | 2074 |

Note: 1. FPKM > 0.1 is the cutoff value for expressed genes.

2. Fisher’s exact test was applied to test for differences in the overall numbers of expressed duplicated genes in WT versus mutant, which showed that significantly more duplicated genes were expressed in the mutant than in WT (*p* value < 2.2e-16).

**Table S4. Statistics of duplicates with CG-only methylation of each duplication category that showed copy-specific expression changes in the *OsMet1-2* mutant versus WT.**

| Duplicate type | All duplicates | | | WGD | Tandem | Proximal | Transposed |
| --- | --- | --- | --- | --- | --- | --- | --- |
| Total gene no. | | 2790 | 1013 | | 242 | 239 | 1296 |
| NH/UL | | 323(11.58%) | 159 | | 16 | 18 | 130 |
| UH/NL | | 203(7.28%) | 65 | | 18 | 12 | 108 |
| UH<UL | | 101(3.62%) | 63 | | 0 | 2 | 36 |
| UH>UL | | 34(1.22%) | 17 | | 2 | 3 | 12 |
| NH/DL | | 61(2.19%) | 26 | | 6 | 4 | 25 |
| DH/NL | | 265(9.50%) | 89 | | 31 | 21 | 124 |
| DH>DL | | 30(1.08%) | 16 | | 4 | 1 | 9 |
| DH<DL | | 29(1.04%) | 18 | | 5 | 0 | 6 |
| UH/DL | | 0(0%) | 0 | | 0 | 0 | 0 |
| DH/UL | | 49(1.76%) | 20 | | 2 | 4 | 23 |
| NH/NL | | 1695(60.75%) | 540 | | 158 | 174 | 823 |

Note: Based on changing direction (up- versus down-regulation) and magnitude (more- versus less) in expression by the two copies of each analyzed duplicate, the duplicated genes that showed expression differences between WT and mutant can be categorized into10 distinct groups (NH/UL, NH/DL, DH/NL, UH/NL, UH < UL, UH > UL, DH > DL, DH < DL, DH/UL and UH/DL; see maintext for definitions). These 10 groups can be combined into two classes according to consequences of the changes with respect to reducing or augmenting expression differences between copies of the duplicates, i.e., convergent and divergent. The duplicated genes that did not show expression difference between WT and mutant are designated as NH/NL.

**Table S5.** Statistics of duplicates with all-context methylation of each duplication category showing copy-specific expression changes in the *OsMet1-2* null mutant versus WT.

| Duplicate type | All duplicates | WGD | Tandem | Proximal | Transposed |
| --- | --- | --- | --- | --- | --- |
| Total gene no. | 3839 | 497 | 437 | 730 | 2175 |
| NH/UL | 279(7.27%) | 57 | 46 | 46 | 130 |
| UH/NL | 428(11.15%) | 47 | 49 | 90 | 242 |
| UH<UL | 59(1.52%) | 16 | 7 | 4 | 32 |
| UH>UL | 27(0.70%) | 11 | 2 | 3 | 11 |
| NH/DL | 64(1.67%) | 10 | 8 | 8 | 38 |
| DH/NL | 619(16.12%) | 57 | 55 | 123 | 384 |
| DH>DL | 41(1.07%) | 11 | 4 | 7 | 19 |
| DH<DL | 37(0.96%) | 10 | 8 | 4 | 15 |
| UH/DL | 13(0.33%) | 5 | 0 | 1 | 7 |
| DH/UL | 51(1.33%) | 12 | 5 | 6 | 28 |
| NH/NL | 2221(57.85%) | 261 | 253 | 438 | 1269 |

Note: Based on changing direction (up- versus down-regulation) and magnitude (more- versus less) in expression by the two copies of each analyzed duplicate, the duplicated genes that showed expression differences between WT and the null mutant can be categorized into10 distinct groups (NH/UL, NH/DL, DH/NL, UH/NL, UH < UL, UH > UL, DH > DL, DH < DL, DH/UL and UH/DL; see maintext for definitions). These 10 groups can be combined into two classes according to consequences of the changes with respect to reducing or augmenting expression differences between copies of the duplicates, i.e., convergent and divergent. The duplicated genes that did not show expression difference between WT and mutant are designated as NH/NL.

**Table S6.** Copy-specific primers for real-time qRT-PCR analysis of 10 randomly selected duplicate gene pairs.

| Pair | Gene | Direction | Sequence | Product size (bp) |
| --- | --- | --- | --- | --- |
| a | LOC_Os01g53880 | Forward | actgctaaaaggctgagggttctaag | 171 |
|  |  | Reverse | cctaaaatggagaatggactggct |  |
|  | LOC_Os05g44810 | Forward | ccatcggttgcttccctacacat | 188 |
|  |  | Reverse | tcctttcttcctctcacctgccata |  |
| b | LOC_Os01g63810 | Forward | aagtatcagaagagattggtgacgca | 127 |
|  |  | Reverse | tcatccacacctacagtcacaccct |  |
|  | LOC_Os05g37450 | Forward | tgggaagacggtgcgtgttagat | 144 |
|  |  | Reverse | cagtcgtgtccttctgaccaatcc |  |
| c | LOC_Os03g26910 | Forward | tcttctttttttatgtgtttctgttcttg | 242 |
|  |  | Reverse | tgaaattaggtggaaatggatcgtat |  |
|  | LOC_Os07g43160 | Forward | atttgtctctttccttttggtccattag | 143 |
|  |  | Reverse | cggtaatagacaccttcaaagccaa |  |
| d | LOC_Os03g42070 | Forward | ggagaggagaaaactcaaactgct | 221 |
|  |  | Reverse | gacattactcccatcgtttttatctg |  |
|  | LOC_Os12g39830 | Forward | tttttagtcagatgtctagggggcg | 119 |
|  |  | Reverse | ggcaatggctcaaagaacctaataaat |  |
| e | LOC_Os03g42070 | Forward | ggagaggagaaaactcaaactgct | 221 |
|  |  | Reverse | gacattactcccatcgtttttatctg |  |
|  | LOC_Os12g39830 | Forward | tttttagtcagatgtctagggggcg | 119 |
|  |  | Reverse | ggcaatggctcaaagaacctaataaat |  |
| f | LOC_Os05g41610 | Forward | ttcagatagtttcaggcttgggag | 185 |
|  |  | Reverse | gatgagcagagcaagaacccg |  |
|  | LOC_Os01g58730 | Forward | atggatttggactcatcgtgtgc | 154 |
|  |  | Reverse | cagagaaacaggtgaatgctggtaat |  |
| g | LOC_Os07g41280 | Forward | gtgtgcgtgtgtaagtaggcggt | 143 |
|  |  | Reverse | tgccaaaacgaccaaaacacact |  |
|  | LOC_Os03g30300 | Forward | ctttttgctgcctatgaagagttgag | 105 |
|  |  | Reverse | acgagcacctgaactgcttactacc |  |
| h | LOC_Os02g07410 | Forward | taaggttgagggtgattcggca | 154 |
|  |  | Reverse | atcgctggttgccttcacactt |  |
|  | LOC_Os06g45670 | Forward | cctaccttcggatctcctccttc | 96 |
|  |  | Reverse | caccctcaaccttcacccactc |  |
| i | LOC_Os02g48660 | Forward | ggaagtcttgctttagcgttttgtt | 127 |
|  |  | Reverse | ttcacagcacgatagcatggagtat |  |
|  | LOC_Os06g21480 | Forward | caaaaacatcagaactcgaacgaat | 89 |
|  |  | Reverse | cctcctgatgattgcgacaactata |  |
| j | LOC_Os02g48660 | Forward | ggaagtcttgctttagcgttttgtt | 127 |
|  |  | Reverse | ttcacagcacgatagcatggagtat |  |
|  | LOC_Os06g21480 | Forward | caaaaacatcagaactcgaacgaat | 89 |
|  |  | Reverse | cctcctgatgattgcgacaactata |  |
| Internal control | Tublin | Forward | taccgtgcccttactgttcc | 234 |
|  |  | Reverse | cggtggaatgtcacagacac |  |

**
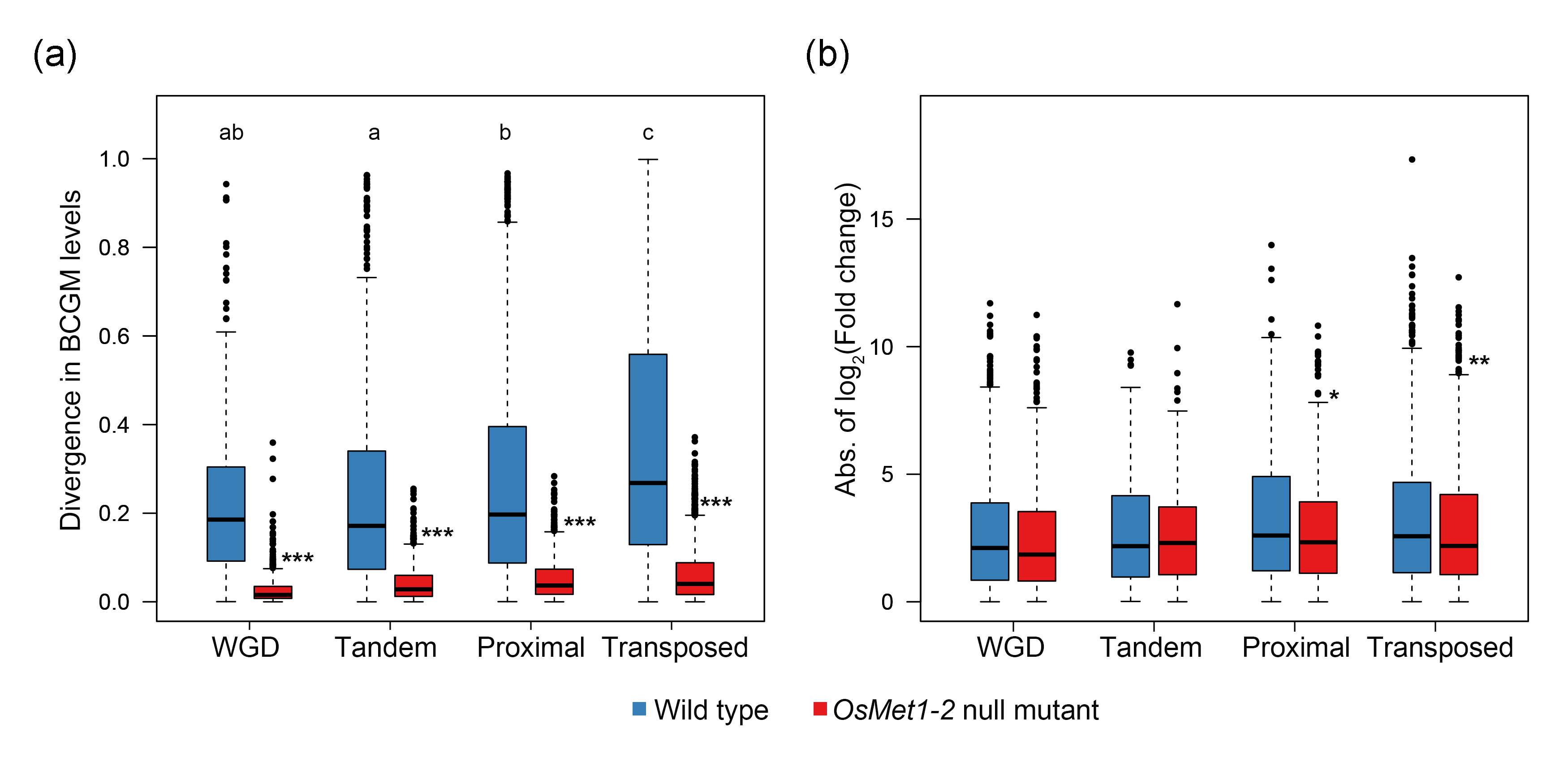
**

**Figure S1. Divergence in body CG methylation (BCGM) level (a) and expression (b) between copies of each of the four categories of duplicates with all-context methylation in WT rice and the *OsMet1-2* null mutant.** The *y* axis in (**a**) denotes divergence in BCGM levels between duplicated copies of each duplication category. Pairwise comparisons showed significant differences in between-copy BCGM divergence among some (indicated by different small letters) but not all (indicated by the same small letters) of the four duplication categories in WT (ANOVA and Tukey’s honestly significant different (HSD) test, *p* < 0.01). Significant reduction of between-copy divergence in BCGM was detected in mutant versus WT in all duplication categories (Kolmogorov-Smirnov test, *p* values<0.01). The *y* axis in (**b**) shows absolute value of fold changes of between-copy expression levels of each duplication category in WT and mutant. Significant reduction of between-copy expression difference in mutant versus WT was detected for proximal and transposed of duplicated genes (Kolmogorov-Smirnov test, *p* values< 0.05).

**
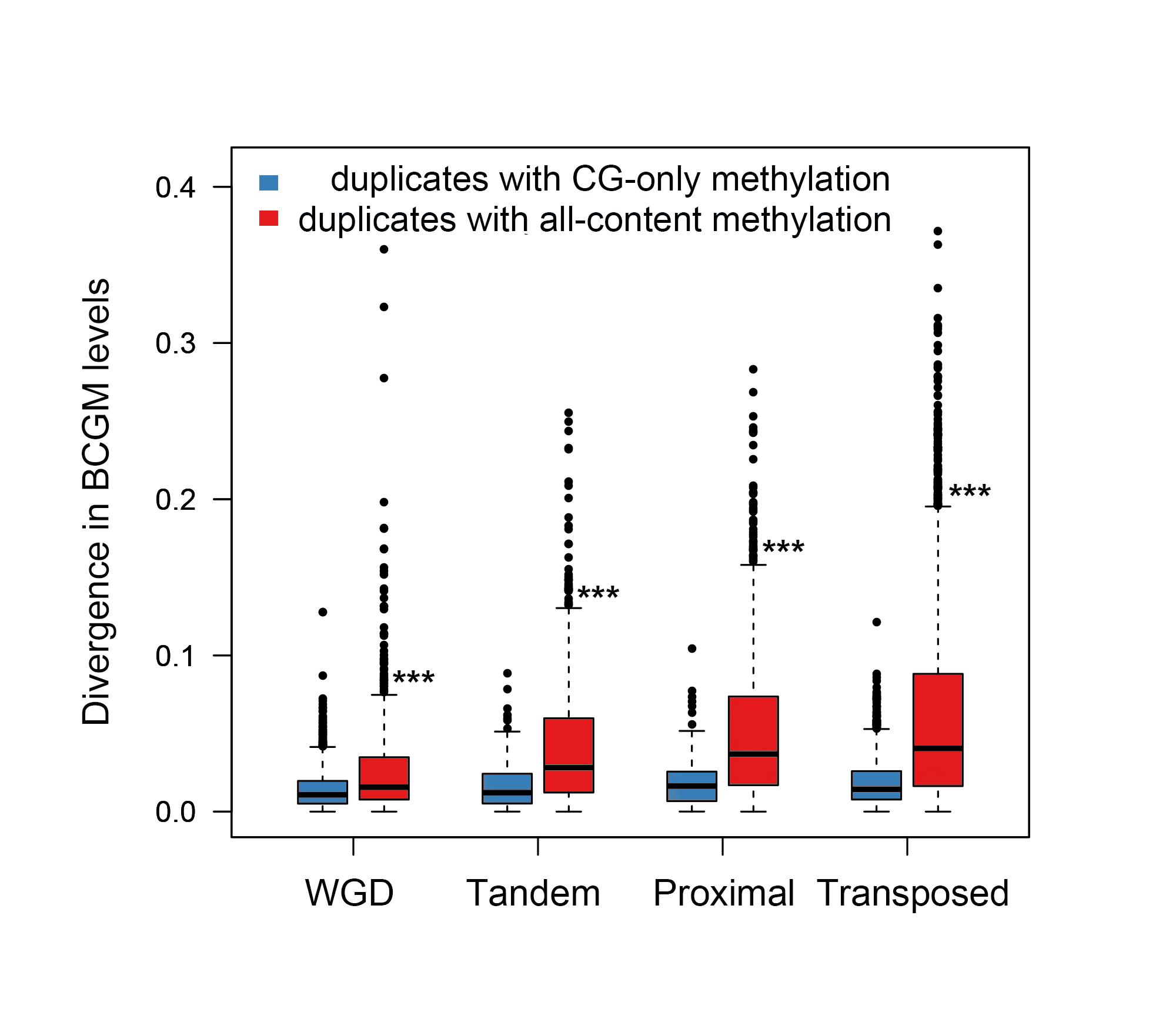
**

**Figure S2. Divergence in body CG methylation (BCGM) level between copies in each of the four categories of duplicates with CG-only methylation and duplicates with all context-methylation in WT rice.** The *y* axis denotes divergence in BCGM levels between copies of each duplication category. Significant difference of between-copy divergence in BCGM was detected in duplicates with CG-only methylation versus duplicates with all context-methylation in all four duplication categories (Kolmogorov-Smirnov test, *p* values<0.01).

**
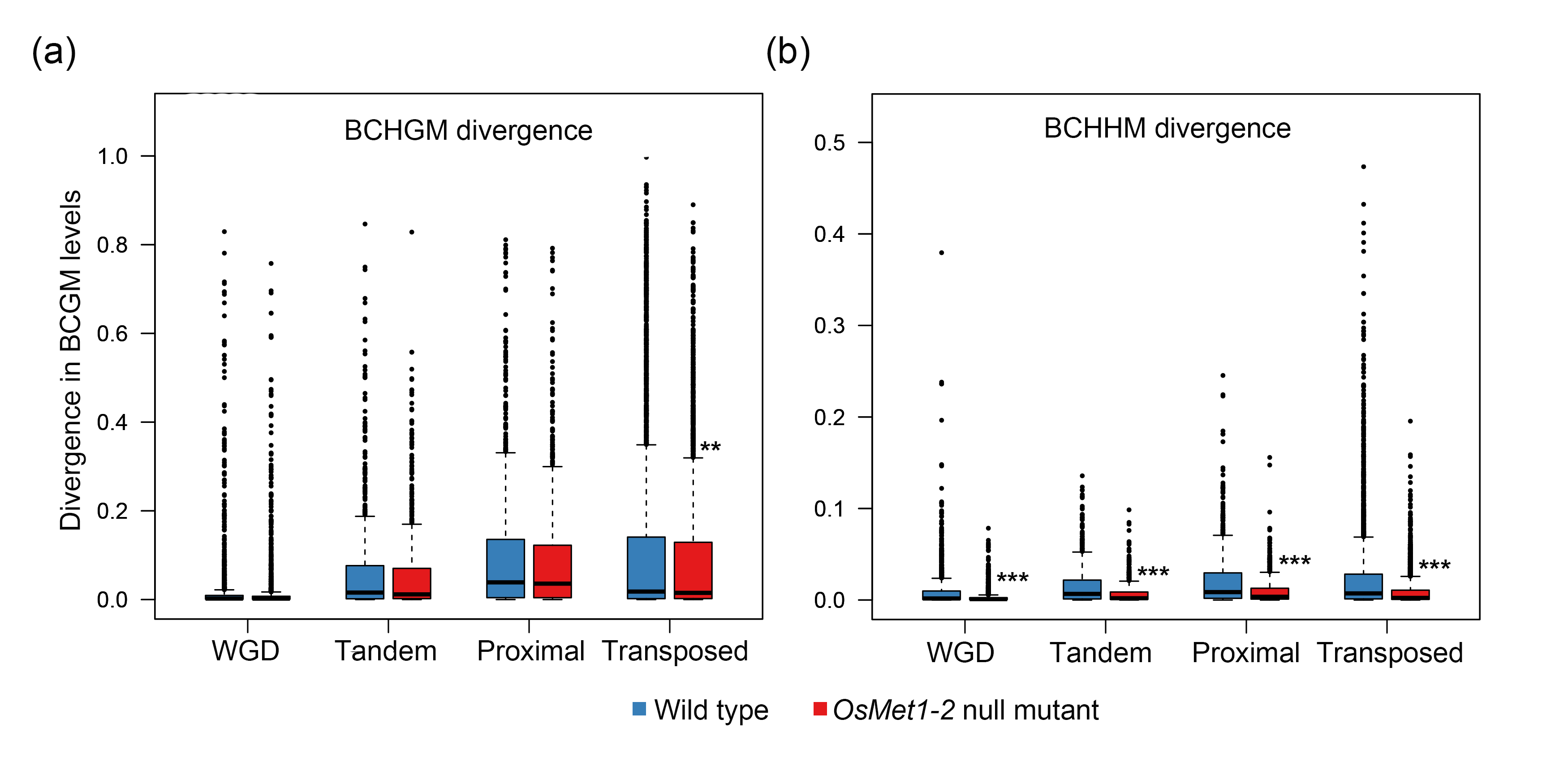
**

**Figure S3. Divergence in body CHG methylation (BCHGM) level (a) and** **body CHH methylation (BCHHM) level (b) between copies in each of the four categories of duplicates in WT and the *OsMet1-2* null mutant.** Except for transposed duplicates, the other three duplicates categories showed no significant BCHGM divergence between WT and mutant. Although BCHHM level was markedly reduced in the mutant compared with WT, it should be noted that the default level of BCHHM is very low in WT, and hence, its reduction is likely inconsequential.

**
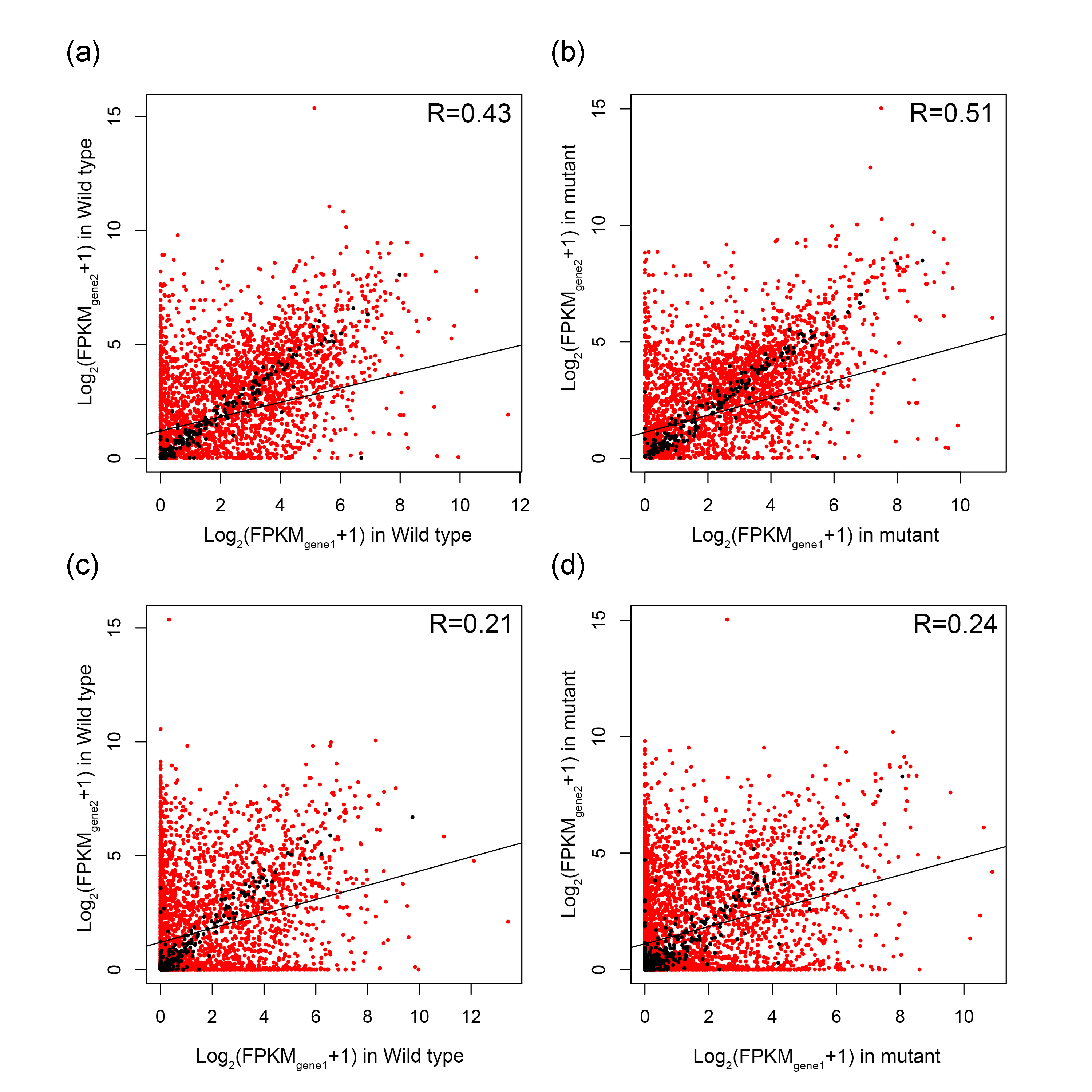
**

**Figure S4. Expression differences of duplicate genes in WT and the *OsMet1-2* null mutant for duplicates with CG-only methylation and duplicates with all-context methylation.** (a**)** duplicates with CG-only methylation in WT; (**b**) duplicates with CG-only methylation in mutant; (c**)** duplicates with all-context methylation in WT; (**d**) duplicates with all-context methylation in mutant. The normalized gene expression (FPKM) in each copy of a given duplicated gene pair was plotted on *x* and *y* axis, respectively. The black lines were linear regressions of expression between the two copies per duplicated gene pair. Red and black dots depict for between-copy differentially (exact condition test, *q* values < 0.05), and equally (*q* values > 0.05) expressed duplicates, respectively, in each genotype.


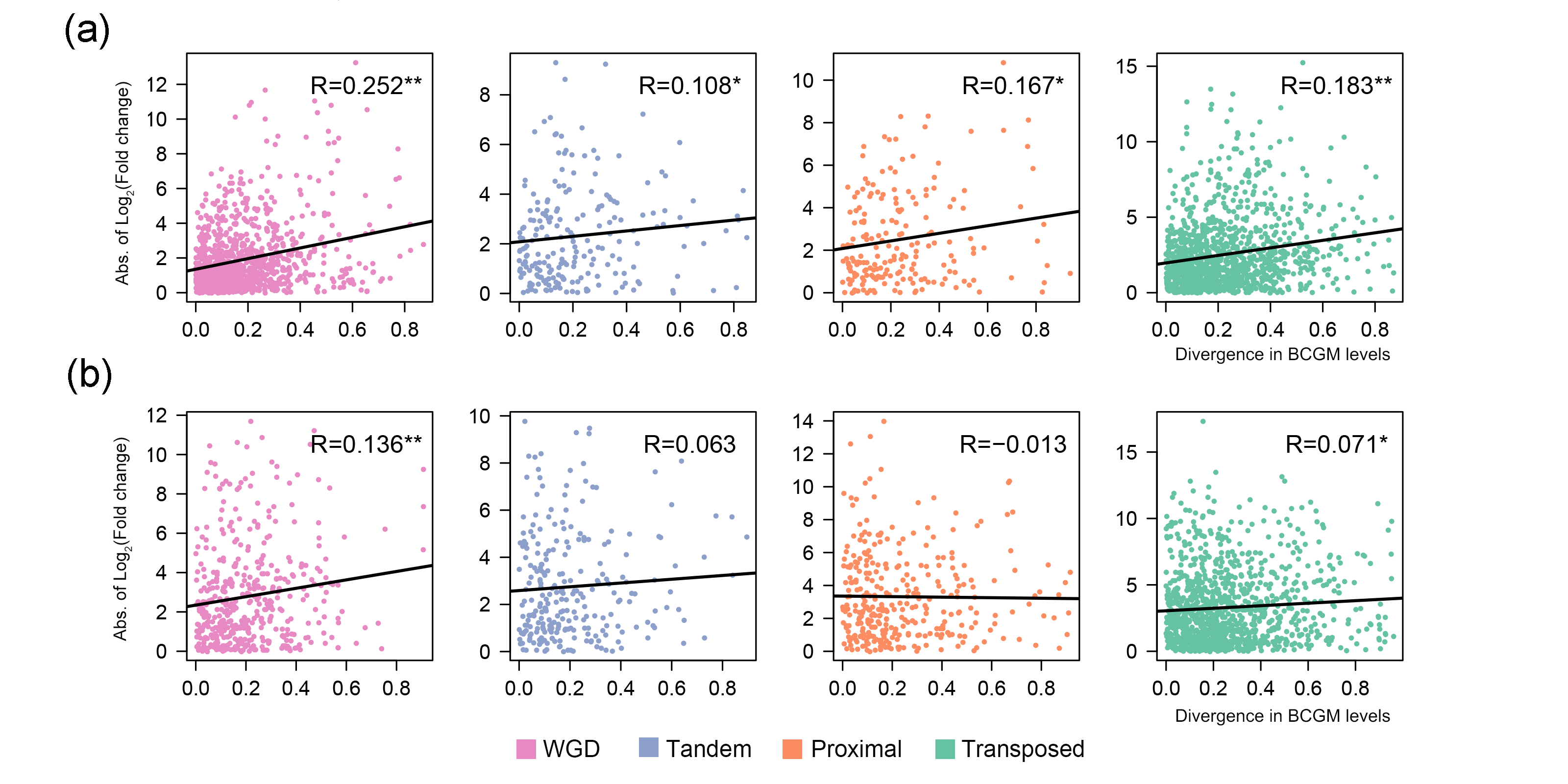


**Figure** **S5. Relationships between body CG methylation (BCGM) difference and expression divergence in duplicates with CG only-methylation (a) and duplicates with all-context methylation (b) in WT rice.** The correlation coefficients were calculated by Pearson's product-moment correlation test. Significant correlations were depicted by asterisks, with * and ** denoting *P* values < 0.05 and 0.01, respectively.


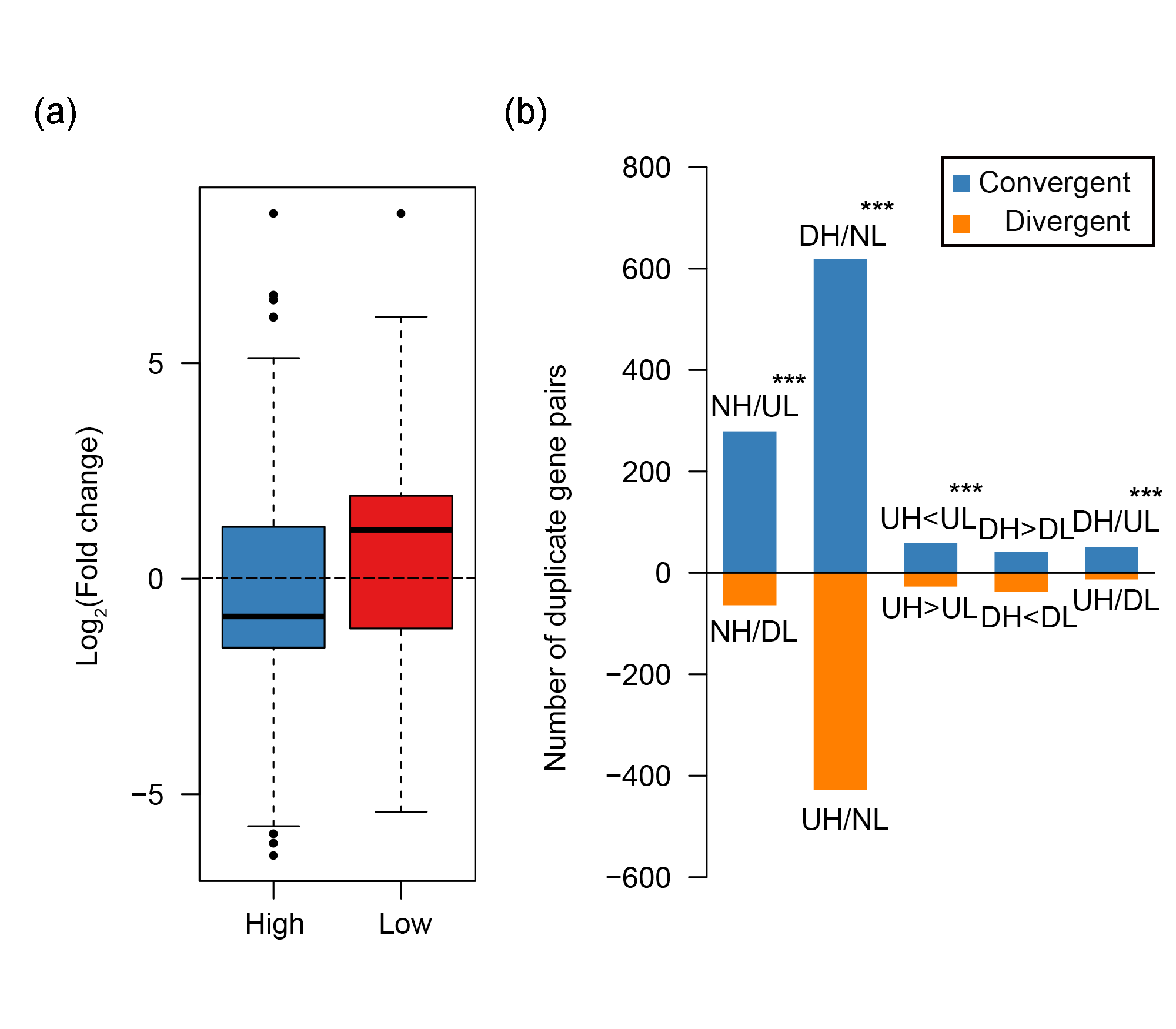


Figure S6. Changes in expression by the higher and lower expression copy-groups, respectively, of all identified rice duplicates with all-context methylation (a), and convergent versus divergent expression changes between copies of the duplicates with all-context methylation (b), as a result of loss of BCGM in the *OsMet1-2* null mutant.

(a) The *y* axis shows fold changes of expression level between WT and mutant by the higher and lower expression copy-groups, respectively, of all identified rice duplicated genes. The dashed black line denotes fold change = 1, which divides the boxplots into two parts. Distribution of gene numbers between the upper and lower parts was tested by binominal exact test, which indicated that the two parts contained significantly different numbers of genes for both the higher expression copy group (*p* value = 5.987e-12) and the lower expression copy-group (*p* value = 6.738e-13). (b) Based on changing direction (up- versus down-regulation) and magnitude (higher- versus lower) in expression by the two copies of each analyzed duplicate, the duplicated genes that showed expression differences between WT and mutant can be categorized into10 distinct groups, which can be combined into two classes according to consequences of the changes with respect to reducing or augmenting expression differences between duplicate copies, i.e., convergent and divergent (see main text for details). The *y* axis shows the number of duplicate genes in each of the 10 groups. A binominal exact test was performed to test for statistical differences in convergence versus divergence in each comparison. The asterisks denote statistical significance: *, ** and *** are *P* values <0.05, 0.01and 0.001, respectively.


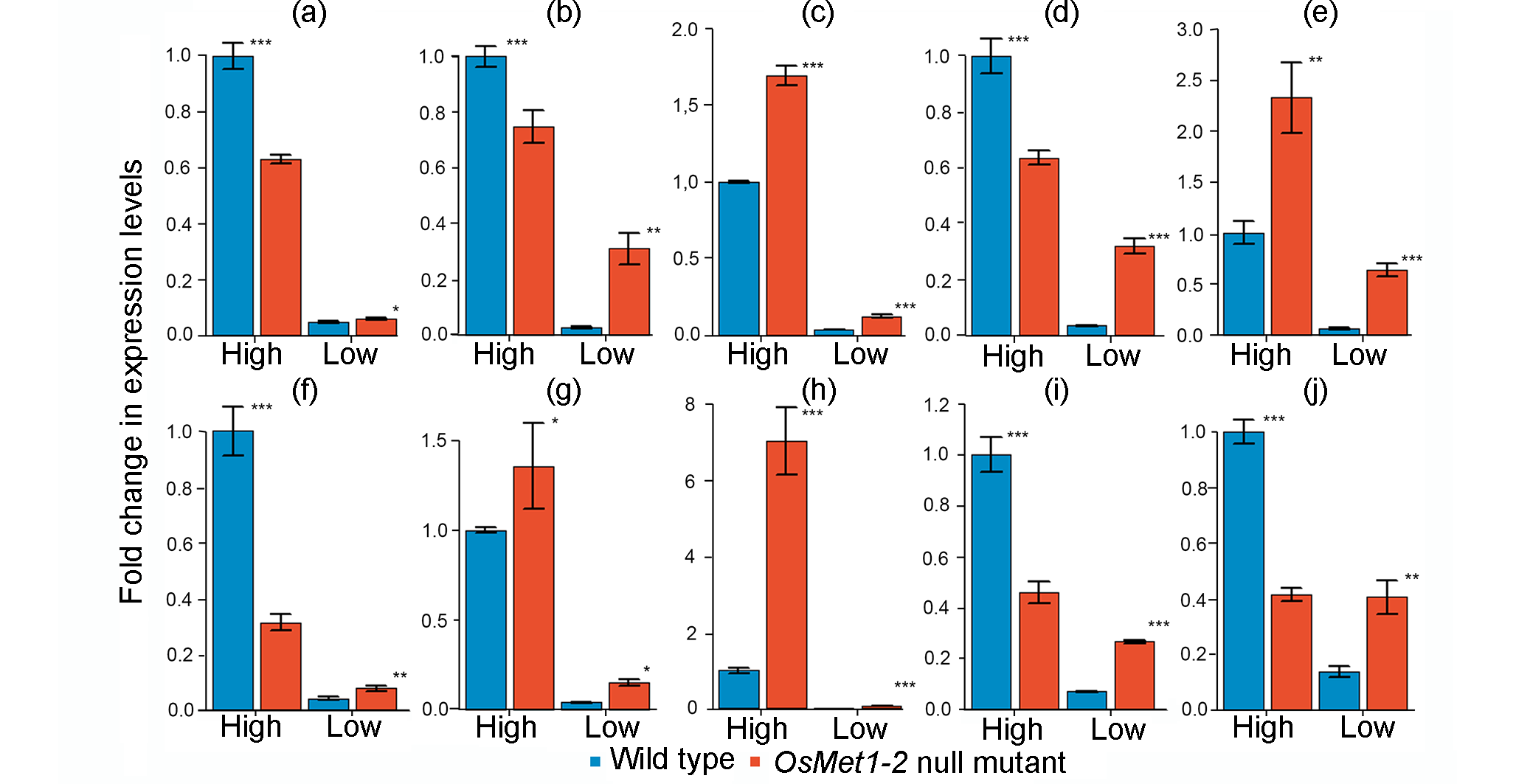


**Figure S7. Validation of expression changes of each copy of a given duplicated gene pair due to loss of gene body CG methylation (BCGM) in the *OsMet1-2* null mutant of rice, by copy-specific real-time qRT-PCR**. For each of the 10 selected duplicate gene pairs (listed in Supplemental Table S3), the means (*y* axis) ± standard deviations (error bar) of the relative expression levels (steady-state transcripts) of the higher- and lower-expression copies in WT (blue) and mutant (red) were presented. The asterisks denote for statistical significance (Student’s t-test): *, ** and *** are *P* values < 0.05, 0.01and 0.001, respectively.

**
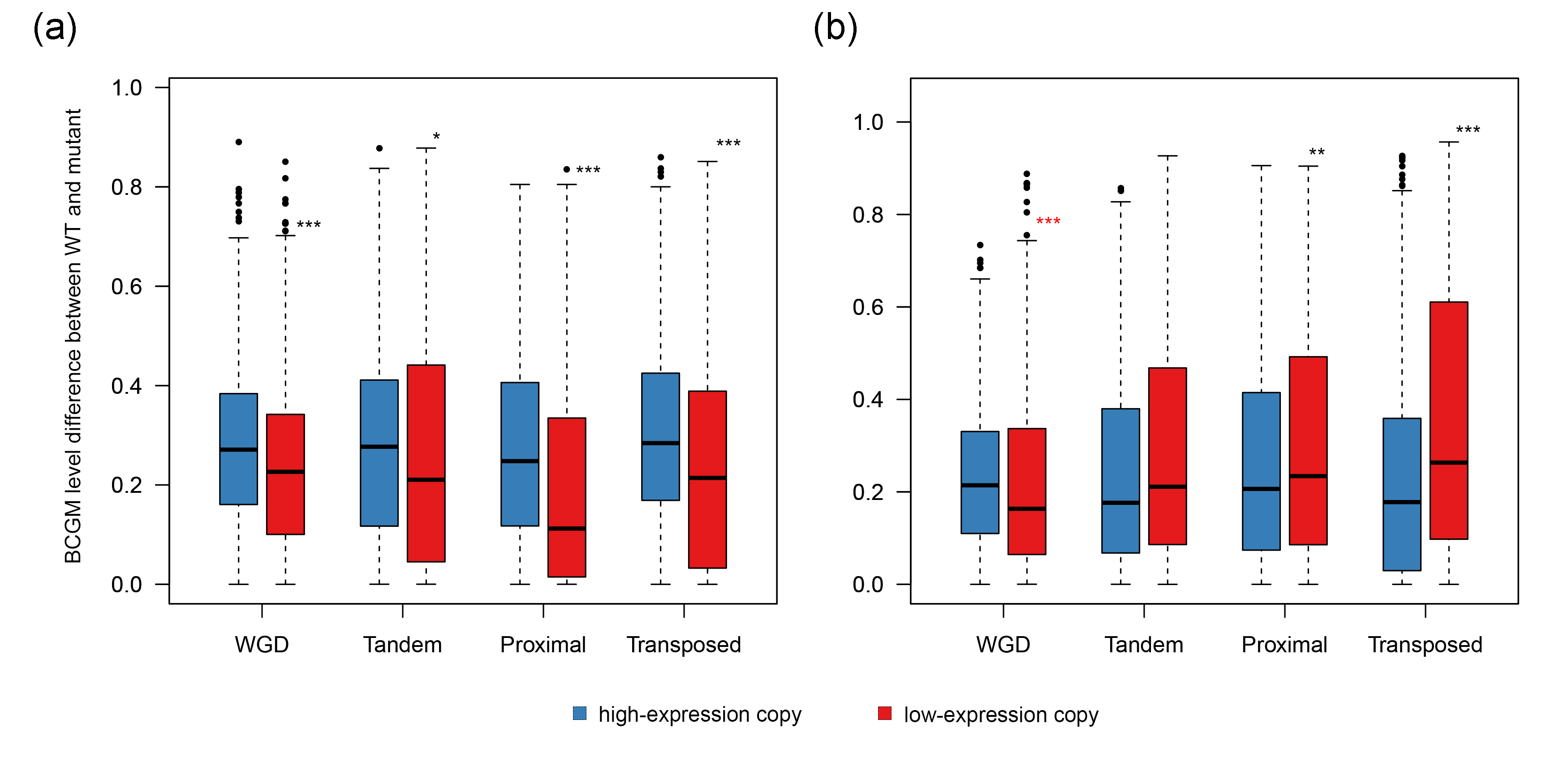
**

**Figure S8. Differences in body CG methylation (BCGM) levels between WT and the *OsMet1-2* null mutant for the higher (blue boxes) and lower (red boxes) expression copies of each duplicate category for duplicates with CG-only methylation (a) and duplicates with all-context methylation (b).** For duplicates with CG-only methylation, the higher-expression copies showed greater loss of BCGM than the lower-expression copies in all four duplication categories (K-S test, *p* value <0.05). By contrast, for duplicates with all-context methylation, there was no consistent pattern of BCGM level difference between the high- versus the low-expression copies in the four duplication categories. The asterisks in red font in (**b**) denote contrasting trend from the rest duplication categories.


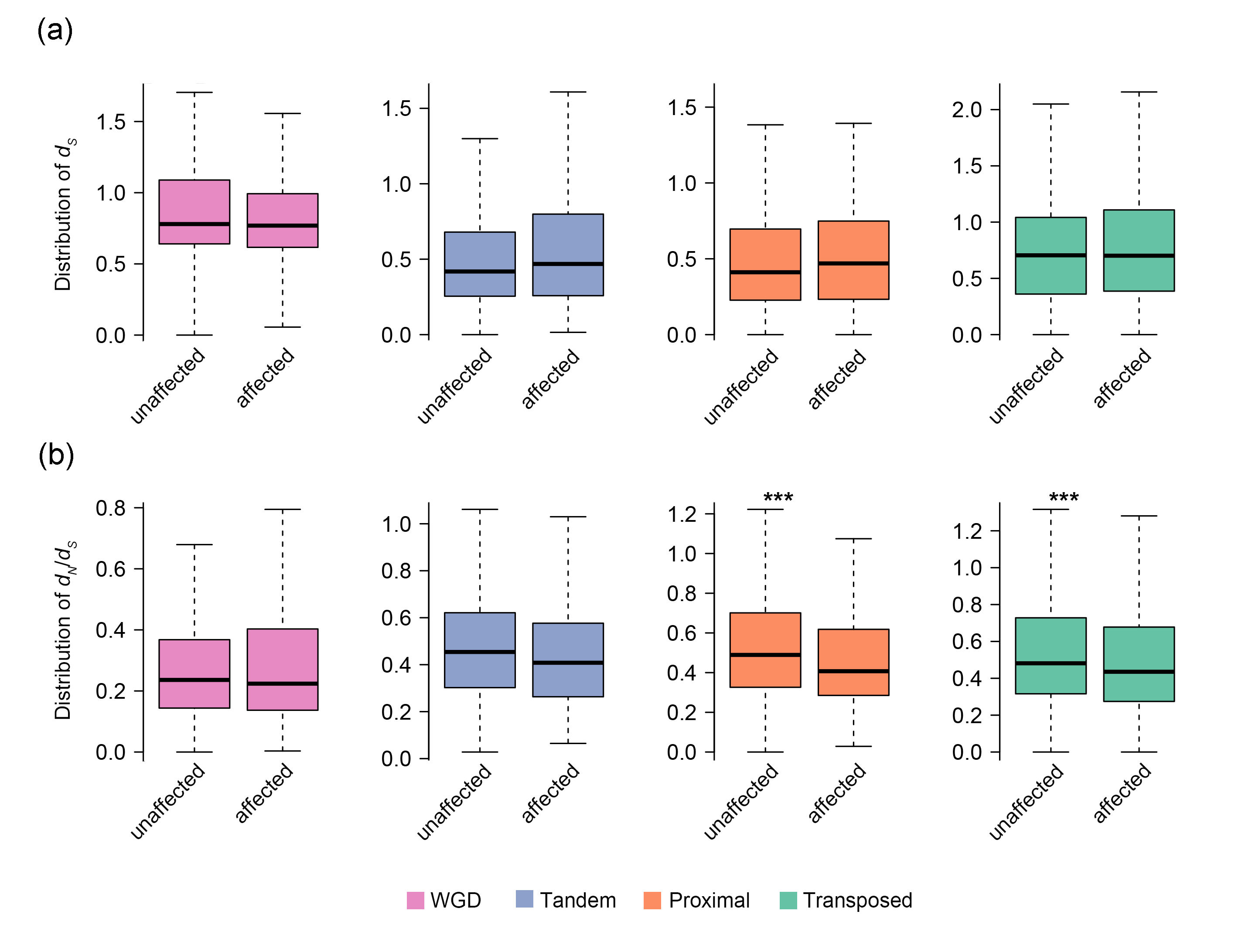


**Figure S9. Comparison of distributions of *d_S_* (a) and *d_N_*/*d_S_* (b) between expression-unaffected and -affected duplicates of each of the four categories with all-context methylation due to loss of BCGM in the mutant.** The Kolmogorov-Smirnov test was conducted for statistical significance. There were no significant changes in *d_S_* distribution for any duplication category (K-S test, *p* values > 0.05), but the ranges of *d_N_*/*d_S_* distribution were significantly larger in expression-unaffected duplicates than expression-affected duplicates (K-S test, *p* values < 0.01) in proximal and transposed duplicates.
